# Pathological underestimation and biomarkers concordance rates in breast cancer patients diagnosed with ductal carcinoma in situ at preoperative biopsy

**DOI:** 10.1038/s41598-022-06206-7

**Published:** 2022-02-09

**Authors:** Hemei Zhou, Jing Yu, Xiaodong Wang, Kunwei Shen, Jiandong Ye, Xiaosong Chen

**Affiliations:** 1 Suzhou Ninth People’s Hospital, 2666 Ludang Road, Wujiang District, Suzhou, 215200 Jiangsu Province China; 2 Comprehensive Breast Health Center, Ruijin Hospital, Shanghai Jiaotong University School of Medicine, 197 Ruijin Er Road, Shanghai, 20025 China

**Keywords:** Cancer, Molecular biology, Biomarkers, Oncology

## Abstract

Ductal carcinoma in situ (DCIS) often upgrade to invasive breast cancer at surgery. The current study aimed to identify factors associated with pathological underestimation and evaluate concordance rates of biomarkers between biopsy and surgery. Patients diagnosed with DCIS at needle biopsy from 2009 to 2020 were retrospectively reviewed. Univariate and multivariate analyses were performed to identify factors associated with pathological underestimation. Concordance rates between paired biopsy samples and surgical specimens were evaluated. A total of 735 patients with pure DCIS at biopsy were included, and 392 patients (53.3%) underwent pathological underestimation at surgery. Multivariate analysis demonstrated that tumor size > 5.0 cm [odds ratio (OR) 1.79], MRI BI-RADS ≥ 5 categories (OR 2.03), and high nuclear grade (OR 2.01) were significantly associated with pathological underestimation. Concordance rates of ER, PR, HER2 status and Ki-67 between biopsy and surgery were 89.6%, 91.9%, 94.8%, and 76.4% in lesions without pathological underestimation, and were 86.4%, 93.2%, 98.2% and 76.3% for in situ components in lesions with pathological underestimation. Meanwhile, in situ components and invasive components at surgery had concordance rates of 92.9%, 93.8%, 97.4%, and 86.5% for those biomarkers, respectively. In conclusion, lesions diagnosed as DCIS at biopsy have a high rate of pathological underestimation, which was associated with larger tumor size, higher MRI BI-RADS category, and higher nuclear grade. High concordances were found in terms of ER, PR, and HER2 status evaluation between biopsy and surgery, regardless of the pathological underestimation.

## Introduction

Ductal carcinoma in situ (DCIS) of the breast accounts for 10–20% of all newly detected breast cancer cases, which is a non-obligate precursor of invasive ductal carcinoma (IDC)^1,2^. Patients diagnosed with DCIS always have a favorable prognosis with a 20-year breast cancer-specific mortality rate of approximately 3.3%^3^.

Several trials were conducted to compare the active surveillance with conventional surgery for low-risk DCIS^4^. However, nearly a quarter of patients diagnosed with pure DCIS at core needle biopsy (CNB) upstaged to IDC at surgery, which is defined as pathological underestimation^5,6^. Carefully selecting eligible patients for watchful waiting and excluding those with an occult invasive component is critical. Meanwhile, pure DCIS has no potential of metastasis, thus lymph node staging at surgery is not routinely indicated^2,7^. But for patients with a pathological underestimation, re-operation for axillary status evaluation is required.

Currently, the systemic individualized treatment of breast cancer is guided by molecular subtype, which was defined according to the expression status of estrogen receptor (ER), progesterone receptor (PR), human epidermal growth factor receptor 2 (HER2), and Ki-67. The molecular phenotype presenting in DCIS could independently predict the disease outcome and invasive recurrence. For some micro-invasion lesion which accounts for only 1% of all breast cancer cases, the small size of the invasive foci makes it difficult to perform the pathological examination. For those patients, whether the pathological interpretation of in situ component could be taken as a reference for the micro-invasive component remains to be studied.

Several studies had reported predictive factors related to pathological underestimation, including clinical, radiological, and histopathological characteristics. However, much less has been reported on the comparison of the status of ER, PR, HER2, and Ki-67 between paired CNB sample and surgical specimen in patients with a final diagnosis of both DCIS and IDC separately^5,7–12^. In the current study, we aim to evaluate the pathological underestimation in patients diagnosed with DCIS at CNB as well as to investigate the accordance rate for biomarkers between paired CNB samples and surgical specimens.

## Results

### Patient population and baseline characteristics

**Figure 1:**
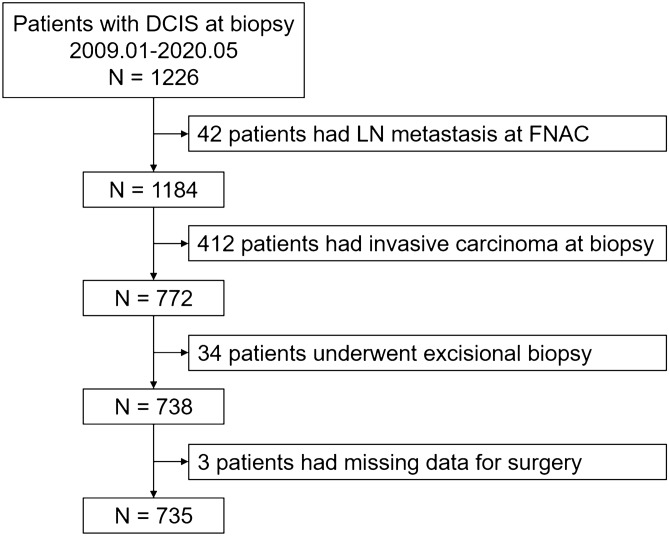
Flow chart. *DCIS* = ductal carcinoma in situ, *LN* lymph node, *FNAC* fine needle aspiration cytology.

A total of 1226 patients diagnosed with DCIS at biopsy were reviewed, and 735 patients were included eventually (Fig. 1). Pure DCIS was confirmed in 343 (46.7%) patients after surgery and the other 392 patients (53.3%) upstaged to IDC (including microinvasion). Baseline characteristics were listed in Table 1. The median age at diagnosis was 52 (range, 24–89) years with 417 patients (56.7%) > 50 years. Over the 718 patients with data of BMI, 561 patients (78.1%) were < 25.00 kg/m^2^. Among all patients, 340 (46.3%) were pre- or peri-menopausal, and 395 (53.7%) were postmenopausal. Totally, 161 patients (21.9%) had benign breast disease history, 36 patients (4.9%) had past malignant tumor history, and 57 (7.8%) patients had a family history of breast cancer. There were 667 patients (90.7%) having a mass at clinical examination, 392 patients (53.3%) having calcification on imaging manifestation, and 99 patients (13.5%) having nipple discharge or erosion as clinical symptom. Regarding the radiological characteristics, 34.8% (245 out of 704) of patients were ≥ 5 BI-RADS category by US, 71.5% (418 out of 585) were ≥ 4B BI-RADS category by MMG, and 32.5% (194 out of 597) were ≥ 5 BI-RADS category by MRI. A total of 691 (94.0%) patients underwent CNB and 44 (6.0%) received VAB. Regarding the pathological characteristics, low or intermediate- grade tumors accounted for 28.2%, high-grade accounted for 16.2%, and the other 409 patients were unknown for the tumor grade. The IHC results showed that 57.3% of patients were ER-positive, 44.8% of patients were PR-positive, 47.5% were HER2-positive, and 48.9% had Ki-67 ≥ 14%.

**Table 1:** Baseline characteristics of 735 patients.

	Total	Surgery of DCIS	Surgery of IDC	*P* value
	N = 735 (%)	N = 343 (%)	N = 392 (%)	
**Age at diagnosis (year)**				0.654
≤ 50	318 (43.3)	145 (42.3)	173 (44.1)	
> 50	417 (56.7)	198 (57.7)	219 (55.9)	
**BMI (kg/m2)**^**a**^				0.320
< 25	561 (78.1)	256 (76.4)	305 (79.6)	
≥ 25	157 (21.9)	79 (23.6)	78 (20.4)	
**Menstrual status**				0.657
Pre-and peri-menopause	340 (46.3)	162 (47.2)	178 (45.4)	
Post-menopause	395 (53.7)	181 (52.8)	214 (54.6)	
**Benign breast disease history**				0.859
Absent	574 (78.1)	269 (78.4)	305 (77.8)	
Present	161 (21.9)	74 (21.6)	87 (22.2)	
**Past malignant tumor history**				0.496
Absent	699 (95.1)	324 (94.5)	375 (95.7)	
Present	36 (4.9)	19 (5.5)	17 (4.3)	
**Family history of breast cancer**				0.407
Absent	678 (92.2)	313 (91.3)	365 (93.1)	
Present	57 (7.8)	30 (8.7)	27 (6.9)	
**Clinical examination of mass**				0.074
Absent	68 (9.3)	39 (11.4)	29 (7.4)	
Present	667 (90.7)	304 (88.6)	363 (92.6)	
**Imaging manifestations of calcification**				0.022
Absent	343 (46.7)	176 (51.3)	167 (42.6)	
Present	392 (53.3)	167 (48.7)	225 (57.4)	
**Nipple discharge or erosion**				1.000
Absent	636 (86.5)	297 (86.6)	339 (86.5)	
Present	99 (13.5)	46 (13.4)	99 (13.5)	
**US BI-RADS**^**b**^				0.011
< 5 category	459 (65.2)	225 (70.3)	234 (60.9)	
≥ 5 category	245 (34.8)	95 (29.7)	150 (39.1)	
**US BI-RADS**^**b**^				0.154
< 4B category	54 (7.7)	30 (9.4)	24 (6.2)	
≥ 4B category	650 (92.3)	290 (90.6)	360 (93.8)	
**MMG BI-RADS**^**c**^				0.015
< 5 category	496 (84.8)	243 (88.7)	253 (81.4)	
≥ 5 category	89 (15.2)	31 (11.3)	58 (18.6)	
**MMG BI-RADS**^**c**^				< 0.001
< 4B category	167 (28.5)	98 (35.8)	69 (22.2)	
≥ 4B category	418 (71.5)	176 (64.2)	242 (77.8)	
**MRI BI-RADS**^**d**^				< 0.001
< 5 category	403 (67.5)	209 (74.6)	194 (61.2)	
≥ 5 category	194 (32.5)	71 (25.4)	123 (38.8)	
**MRI BI-RADS**^**d**^				0.316
< 4B category	38 (6.4)	21 (7.5)	17 (6.2)	
≥ 4B category	559 (93.6)	259 (92.5)	259 (93.8)	
**Tumor size (cm)**				< 0.001
≤ 2.0	156 (21.2)	97 (28.3)	59 (15.1)	
2.1–5.0	463 (63.0)	208 (60.6)	255 (65.1)	
> 5.0	116 (15.8)	38 (11.1)	78 (19.9)	
**Biopsy method**				0.028
CNB	691 (94.0)	315 (91.8)	376 (95.9)	
VAB	44 (6.0)	28 (8.2)	16 (4.1)	
**Nuclear grade at biopsy**				0.005
Low/intermediate	207 (28.2)	116 (33.8)	91(23.2)	
High	119 (16.2)	55 (16.0)	64 (16.3)	
Unknown	409 (55.6)	172 (50.1)	237 (60.5)	
**ER at biopsy**^**e**^				0.631
Negative	273 (42.7)	124 (41.6)	149 (43.7)	
Positive	366 (57.3)	174 (58.4)	192 (56.3)	
**PR at biopsy**^**f**^				0.056
Negative	353 (55.2)	152 (51.0)	201 (58.8)	
Positive	287 (44.8)	146 (49.0)	141 (41.2)	
**HER2 at biopsy**^**g**^				0.391
Negative	145 (22.4)	70 (23.3)	75 (21.7)	
Positive	307 (47.5)	134 (44.7)	173 (50.0)	
Uncertain	194 (30.0)	96 (32.0)	98 (28.3)	
**Ki-67 at biopsy**^**h**^				0.058
< 14%	328 (51.1)	164 (55.2)	164 (47.5)	
≥ 14%	314 (48.9)	133 (44.8)	181 (52.5)	

^a^17 cases unknown in BMI.

^b^29 cases unknown in US BI-RADS.

^c^150 cases unknown in MMG BI-RADS.

^d^138 cases unknown in MRI BI-RADS.

^e^96 cases unknown in ER at biopsy.

^f^95 cases unknown in PR at biopsy.

^g^89 cases unknown in HER2 at biopsy.

^h^93cases unknown in Ki-67 at biopsy.

*P* values were estimated using Fisher’s exact tests; unknown categories were excluded from *P* value estimation.

*DCIS* ductal carcinoma in situ, *IDC* invasive ductal carcinoma, *BMI* body mass index, *US BI-RADS* Ultrasound Breast Imaging Reporting and Data System, *MMG BI-RADS* Mammography Breast Imaging Reporting and Data System, *MRI BI-RADS* Magnetic Resonance Imaging Breast Imaging Reporting and Data System, *CNB* core needle biopsy, *VAB* vacuum-assisted biopsy, *ER* estrogen receptor, *PR* progesterone receptor, *HER2* human epidermal growth factor Receptor 2, *Ki-67* Proliferation index.

### Characteristics associated with pathological underestimation

Univariate analysis demonstrated that Imaging manifestations of calcification (57.4% vs. 48.7%, *P* = 0.022), US BI-RADS ≥ 5 (39.1% vs. 29.7%, *P* = 0.011), MMG BI-RADS ≥ 4B (77.8% vs. 64.2%, *P* < 0.001), MRI BI-RADS ≥ 5 (38.8% vs. 25.4%, *P* < 0.001), larger tumor size (*P* = 0.001), and higher nuclear grade at biopsy (*P* = 0.005) were significantly correlated with pathological underestimation. Furthermore, patients underwent pathological underestimation were more likely to be biopsied by CNB, with the underestimate rate 54.4% in the CNB cohort and 36.4% in the VAB cohort, respectively (*P* = 0.028). Regarding the IHC assessment at biopsy, PR negativity (58.8% vs. 51.0%, *P* < 0.001) and Ki-67 ≥ 14% (52.5% vs. 44.8%, *P* = 0.058) were marginally significant associated with pathological underestimation (Table 1).

In multivariate analysis, MRI BI-RADS ≥ 5 (vs MRI BI-RADS < 5, OR 2.03, 95% CI 1.30–3.15, *P* = 0.002), tumor size (> 2.0 cm vs ≤ 2.0 cm, OR 1.94, 95% CI 0.93–4.08, *P* = 0.079; > 5.0 cm vs ≤ 2.0 cm, OR 1.79, 95% CI 1.06–3.02, *P* = 0.028), and nuclear grade at biopsy (high vs low/intermediate, *P* = OR = 2.01, 95% CI 1.27–3.18, *P* = 0.003) remained significantly associated with pathological underestimation. Whereas, calcification, US BI-RADS category, MMG BI-RADS category, biopsy method, PR status, and Ki-67 results at biopsy were no longer significant in multivariate analysis (*P* > 0.05, Table 2).

**Table 2:** Multivariable analysis of baseline characteristics for pathological underestimation.

	OR	95%CI	*P* value^a^
Imaging manifestations of calcification (Present vs. Absent)	0.81	0.65–1.73	0.809
US BI-RADS (≥ 5 category vs. < 5 category)	0.86	0.54–1.36	0.520
MMG BI-RADS (≥ 4B category vs. < 4B category)	0.82	0.51–1.33	0.424
**MRI BI-RADS (≥ 5 category vs. < 5 category)**	2.03	1.30–3.15	**0.002**
**Tumor size (cm)**			0.075
2.1–5.0 vs. ≤ 2.0	1.94	0.93–4.08	0.079
> 5.0 vs. ≤ 2.0	1.79	1.06–3.02	**0.028**
**Nuclear grade at biopsy**			0.011
High vs. Low/ intermediate	2.01	1.27–3.18	**0.003**
Unknown vs. Low/ intermediate	1.55	0.86–2.80	0.142
Biopsy method (VAB vs. CNB)	0.81	0.28–2.32	0.691
PR at biopsy (Positive vs. Negative)	0.98	0.65–1.48	0.913
Ki-67 at biopsy (≥ 14% vs. < 14%)	1.07	0.70–1.62	0.767

*P* values were estimated using Binary logistic regression (backward LR).

*OR* Odds Ratios, *CI* confidence interval, *US BI-RADS* Ultrasound Breast Imaging Reporting and Data System, *MMG BI-RADS* Mammography Breast Imaging Reporting and Data System, *MRI BI-RADS* Magnetic Resonance Imaging Breast Imaging Reporting and Data System, *CNB* core needle biopsy, *VAB* Vacuum-assisted biopsy, *ER* estrogen receptor, *PR* progesterone receptor, *HER2* human epidermal growth factor Receptor 2, *Ki-67* Proliferation index;

Significant values are in [bold].

### Concordance between CNB and surgery for biomarkers in patients without pathological underestimation

**Figure 2:**
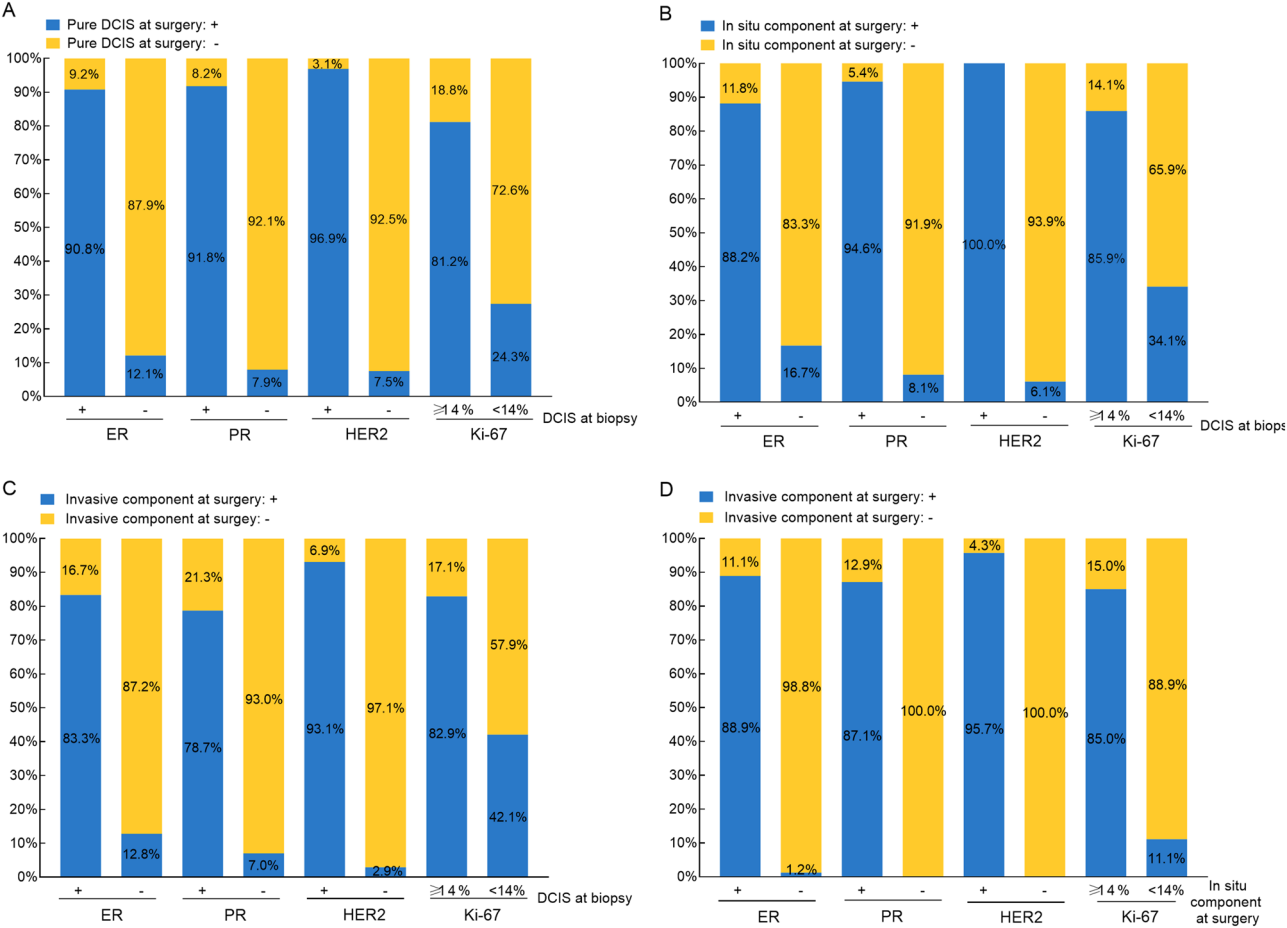
Biomarkers concordance rates between CNB and surgery. (**A**) Concordance rates for ER, PR, HER2, and Ki-67 between DCIS at CNB and surgery in patients without pathological underestimation. (**B**) Concordance rates for ER, PR, HER2, and Ki-67 between DCIS at CNB and in situ components at surgery in patients with pathological underestimation. (**C**) Concordance rates for ER, PR, HER2, and Ki-67 between DCIS at CNB and invasive components at surgery in patients with pathological underestimation. (**D**) Concordance rates for ER, PR, HER2, and Ki-67 between in situ components and invasive components at surgery in patients with pathological underestimation. *DCIS* ductal carcinoma in situ, *CNB* core needle biopsy, *ER* estrogen receptor, *PR* progesterone receptor, *HER2* human epidermal growth factor receptor 2, *Ki-67* proliferation index.

Among 342 patients remaining DCIS after surgery, there were 45 patients with unknown ER, PR status, 175 patients with unknown or uncertain in HER2 status, and 48 patients having unknown results for the Ki-67. Evaluation of ER status between biopsy and surgery had a concordance rate of 89.6%, with a good overall agreement (κ = 0.786). The concordance rates for PR and HER2 were 91.9% and 94.8%, with a κ value of 0.839 and 0.886, respectively. When comparing with lesions at biopsy, there were more Ki-67 highly expressed lesions detected at surgery, and the concordance rate was 76.4% (κ = 0.530, Table 3, Fig. 2A).

**Table 3:** Concordance between biopsy and surgery for biomarkers in patients without pathological underestimation.

DCIS at biopsy	DCIS at surgery		Concordance rate (%)	Kappa	*P* value
	Negative (Ki-67 < 14%)	Positive (Ki-67 ≥ 14%)			
**ER**^**a**^			89.6	0.786	< 0.001
Negative	109	15			
Positive	16	158			
**PR**^**a**^			91.9	0.839	0.001
Negative	140	12			
Positive	12	134			
**HER2**^**b**^			94.8	0.886	< 0.001
Negative	37	3			
Positive	4	124			
**Ki-67**^**c**^			76.4	0.530	< 0.001
< 14%	119	45			
≥ 14%	25	108			

^a^45 cases unknown in ER PR.

^b^175 cases unknown or uncertain in HER2.

^c^48 cases unknown in Ki-67.

Κ values were estimated using kappa test.

*DCIS* ductal carcinoma in situ, *ER* estrogen receptor, *PR* progesterone receptor, *HER2* human epidermal growth factor Receptor 2, *Ki-67* Proliferation index.

When categorized into five subtypes, 13.2%, 9.0%, 19.8%, 56.3%, and 1.8% of the patients were categorized as Luminal A-like, Luminal B-like (HER2-negative), luminal B-like (HER2-positive), HER2-positive (non-luminal), and triple-negative at biopsy, respectively. There were 13 out of 32 patients (36.4%) with Luminal B-like (HER2-positive) breast cancer at biopsy classified as other subtypes at surgery. Whereas, only and 8 out of 94 patients (12.8%) with HER2-positive (non-luminal) lesions had different molecular subtypes at surgery. Overall, the concordance rate was 80.2% with a good agreement (κ value = 0.684) between biopsy and surgery (Table S1).

Photographs for discordant IHC results in terms of PR, HER2, and Ki-67 in patients without pathological underestimation were shown in Figure S1.

### Concordance between biopsy and surgery for biomarkers in patients with pathological underestimation

There were 392 patients upstaged to IDC at surgery, among which 212 had IHC results for in situ component. Patients with unknown results for IHC were excluded from the analysis.

Regarding in situ component in IDC + DCIS lesions, concordance rates were 86.4% for ER status, 93.2% for PR status, 98.2% for HER2 status, and 76.3% for Ki-67 result, respectively (Fig. 2B). And the overall agreements were also good for receptor status (ER: κ = 0.712, PR: κ = 0.864, HER2: κ = 0.957) and moderate for Ki-67 results (κ = 0.522, Table 4). The molecular subtype had concordance rate of 72.9%, with only good agreement (κ value = 0.611). HER2 positive (non-luminal) DCIS had a concordance rate of 86.5% (90 out of 104 patients) between biopsy and surgery, which ranked the highest among five molecular subtypes. Luminal A-like DCIS only had a concordance rate of 63.3% (19 out of 0 patients, Table S2).

**Table 4:** Concordance between CNB and surgery for biomarkers in patients with pathological underestimation.

DCIS at biopsy	In Situ Components at surgery					Invasive Components at surgery				
	Negative (Ki-67 < 14%)	Positive (Ki-67 ≥ 14%)	Concordance rate (%)	Kappa	*P* value	Negative (Ki-67 < 14%)	Positive (Ki-67 ≥ 14%)	Concordance rate (%)	Kappa	*P* value
**ER**^**a**^			86.4	0.712	< 0.001			85.0	0.699	< 0.001
Negative	60	12				130	19			
Positive	14	105				32	160			
**PR**^**b**^			93.2	0.864	< 0.001			87.1	0.730	< 0.001
Negative	91	8				187	14			
Positive	5	87				30	111			
**HER2**^**c**^			98.2	0.957	< 0.001			94.3	0.870	< 0.001
Negative	31	2				67	2			
Positive	0	81				11	148			
**Ki-67**^**d**^			76.3	0.522	< 0.001			71.0	0.412	< 0.001
< 14%	60	31				95	69			
≥ 14%	14	85				31	150			

^a^51 cases unknown in ER at biopsy; 150 cases unknown in ER at In Situ Component at surgery of IDC.

^b^51 cases unknown in PR at biopsy; 150 cases unknown in PR at In Situ Component at surgery of IDC.

^c^144 cases unknown or uncertain in HER2 at biopsy; 170 cases uncertain in HER2 at Invasive Component at surgery of IDC; 233 cases unknown or uncertain in HER2 at In Situ Component at surgery of IDC.

^d^47 cases unknown in Ki-67 at Invasive Component at surgery of IDC; 155 cases unknown in Ki-67 at In Situ Component at surgery of IDC.

κ values were estimated using kappa test.

*DCIS* ductal carcinoma in situ, *IDC* invasive ductal carcinoma, *ER* estrogen receptor, *PR* progesterone receptor, *HER2* human epidermal growth factor Receptor 2, *Ki-67* Proliferation index.

Evaluation of ER, PR, HER2, and Ki-67 at biopsy had a concordance rate of 85.0%, 87.1%, 94.3%, 71.0% with the invasive component at surgery (Fig. 2C). The overall agreements were good for receptor status (κ = 0.699, κ = 0.730, and κ = 0.870, respectively) and moderate for Ki-67 results (κ = 0.412, Table 4). The molecular subtype had a good agreement with a κ value of 0.635, and the concordance rate between biopsy and surgery was 75.0%. Among the 11 patients diagnosed with luminal B-like (HER2-negative) DCIS at biopsy only 21 (63.6%) remained the same molecular subtype at surgery. HER2-positive (non-luminal) lesions showed the highest concordance rate (86.3%, 44 out of 51 patients) among five subtypes (Table S3).

In patients who underwent pathological underestimation, discordance in IHC results was shown in Figure S2.

### Concordance between in situ and invasive component for biomarkers in IDC + DCIS patients

A total of 212 patients were compared for the in situ and the invasive component of the same tumor tissue in terms of ER, PR, and Ki-67. And HER2 status was compared after excluding an additional 60 patients with uncertain results for HER2 expression. The concordance rate was 92.9% for ER status, 93.8% for PR status, 97.4% for HER2 status, and 86.5% for the Ki-67 (Fig. 2D). And the above biomarkers all showed good agreement, with κ values of 0.857, 0.875, 0.945, and 0.723, respectively (Table 5). As for the molecular subtype, the in situ and the invasive component also showed good agreement (κ = 0.820) with a concordance rate of 87.5%. Of note, for the 7 patients diagnosed with triple negative breast cancer, the concordance rate between in situ component and invasive component was 100%. However, the in-situ component classifying as Luminal B-like (HER2-positive) only had a concordance rate of 71.9% (23 out of 32) with the invasive component (Table S4).

**Table 5:** Concordance between in situ and invasive component for biomarkers in IDC + DCIS patients.

In Situ components	Invasive components		Concordance rate (%)	Kappa	*P* value
	Negative	Positive			
	(Ki-67 < 14%)	(Ki-67 ≥ 14%)			
**ER**^**a**^			92.9	0.857	< 0.001
Negative	85	1			
Positive	14	112			
**PR**^**b**^			93.8	0.875	< 0.001
Negative	109	0			
Positive	13	88			
**HER2**^**c**^			97.4	0.945	< 0.001
Negative	59	0			
Positive	4	89			
**Ki-67**^**d**^			86.5	0.723	< 0.001
< 14%	72	9			
≥ 14%	19	108			

^a^150 cases unknown in ER.

^b^150 cases unknown in PR.

^c^170 cases uncertain in HER2 at Invasive Component at surgery of IDC; 233 cases unknown or uncertain in HER2 at In Situ Component at surgery of IDC.

^d^155 cases unknown in Ki-67.

Κ values were estimated using kappa test.

*DCIS* ductal carcinoma in situ, *DCIS* + *IDC* invasive ductal carcinoma with in situ ductal carcinoma component, *ER* estrogen receptor, *PR* progesterone receptor, *HER2* human epidermal growth factor Receptor 2, *Ki-67* Proliferation index.

The Figure S3 showed the discordant IHC results in terms of ER in synchronous DCIS and IDC within a surgery sample.

## Discussion

In the current study, we included 735 patients with DCIS at CNB and demonstrated that pathological underestimation was independently associated with tumor size, MRI BI-RADS category, and nuclear grade at CNB. Good concordances were observed between CNB and surgery in terms of ER, PR, and HER2 status irrespective of the presence of pathological underestimation or not. Whereas, there were more Ki-67 highly expressed lesions detected at surgery compared with at CNB. Regarding lesions with DCIS and IDC components, the overall agreement was also good for ER, PR, HER2, and Ki67 analysis between in situ and invasive components.

The majority of the patients (94.0%) in our center were biopsied with 14-G CNB, and the rate of pathological underestimation was 53.3%. However, previous studies reported that the underestimation rate was approximately 15–20% by using the VAB^13,14^. The method of the biopsy would influence the presence of pathological underestimation. Kim reported that the underestimation rate was 49.8% for the CNB and 29.2% for the vacuum method (*P* < 0.001)^8^. And in Park’s study, the underestimate rate was 50.0% and 18.8%, respectively (*P* < 0.001)^15^. Our result was consistent with previous data for the CNB cohort, and univariate analysis also showed that the underestimate rate was lower in patients who received VAB than CNB. Indeed, VAB by using Mammotome (8-gauge) and Mammotome elite (13-gauge) could provide more tissue samples, which can increase the diagnostic accuracy of biopsy. However, the Mammotome is not covered by the Chinese health insurance or government, and the Mammotome elite was just covered by the health care program at the end of the year 2018. Thus, when considering the treatment cost, both surgeons and patients may prefer 14-gauge CNB over VAB. Furthermore, using stereotactic biopsies instead of sonographic guidance may lower the risk of pathological underestimation (21.8% vs. 39.9%)^5^. It was acknowledged that DCIS is always associated with microcalcifications but not mass-like lesions. As was reported, mammography screening could increase the recall rate of suspicious microcalcifications, as well as the incidence of DCIS. However, there was lack of mammography national screening programs in China, thus the most common complaint was breast lump. In the current study, nearly 90% of the patients had mass-like lesions, thus we used the ultrasound-guided biopsy to diagnosis breast disease. Notably, even with an underestimation rate of 15%, it is still not appropriate to use the pathological results of biopsy specimen as a definitive diagnosis, as well as to safely guide the active surveillance or omit the axillary surgery. Therefore, identifying patients who are at high risk of pathological underestimation is of great importance.

Several factors were reported to be associated with pathological underestimation in previous studies, including palpability, high nuclear grade, BI-RADS category 5, HER2 positivity, Ki-67 overexpression, suspicious invasion, mammographic mass finding, and radiological tumor size ≥ 2.0 cm^5,7–12^. In the current study, we found that MRI BI-RADS ≥ 5, maximum mass diameter > 5.0 cm, and high nuclear grade were independent predictors for pathological underestimation. The presence of high nuclear grade and at CNB have been shown to be associated with an invasive component at surgical specimen^5,16^. Meanwhile, it has also been reported that high-grade DCIS developed to the invasive tumor more quickly than low-grade lesions^17^. This may indicate that high-grade DCIS was at a more advanced stage during progression, thus was more likely to harbor co-existing invasive component. Regarding the imaging feature, previous studies have suggested that breast MRI was highly sensitive for detecting and evaluating breast cancer of various types. DCIS often manifests as non-mass-like enhancements with segmental or ductal distribution, and presents clumped internal architecture in the MRI imaging^14,18–21^. The common manifestation of DCIS included lump as well as segmental distribution in the current study. Kim et al. reported that tumor size > 2.0 cm was an independent predictor of underestimation, which was consistent with the present findings^8^. A plausible reason may be that the larger target area for sampling increases the possibility of sampling error^11^. This suggests that increasing the amount of sample collection by using a thicker needle or increasing the number of cores examined may help to improve the diagnostic accuracy for large tumors.

Treatment patterns of breast cancer, including DCIS, relies on molecular subtype, which was determined by ER, PR, HER2 status, and Ki-67 index^22–26^. However, unlike invasive breast cancer, the IHC assessment of biomarker expression is less performed for DCIS at CNB. In the current study, in patients with a final diagnosis of DCIS at surgery, we observed good concordance rates regarding ER (89.6%), PR (91.9%), and HER2 (94.8%) status. Whereas, Ki-67 only showed a moderate agreement. Similar results had been constantly reported for invasive carcinoma. Tamaki et al. reported that CNB can provide reliable information on ER (κ = 0.82), PR (κ = 0.66), and HER2 (κ = 0.64) status of patients^26^. A meta-analysis study also demonstrated the high diagnostic accuracy of CNB in evaluating ER, PR, and HER2 status compared with open excision biopsy in breast cancer patients^27^. The poor agreement in terms of Ki-67 had also been recorded for invasive breast cancer^28^, which may due to the poorer fixation of surgical specimens compared with CNB ones and intra-tumor heterogeneity^29^. Meanwhile, the wound response of biopsy may also cause an increase in Ki-67 expression, since the invasive diagnostic procedure may accelerate the tumor growth^30^. Another point is the highly inconsistent measurement of Ki-67 among testing laboratories and pathologists, and further efforts were needed to set universally recognized cutoff for this biomarker.

In patients with a pathological underestimation, both the in situ components and the invasive components in surgery specimens showed good agreements (κ > 0.6) with CNB samples in terms of ER, PR, and HER2 status, which has rarely been evaluated in previous studies. Additionally, high concordance rates were also observed in terms of ER (92.9%), PR (93.8%), HER2 (97.4%), Ki-67 (86.5%) between the invasive component and in situ component at the surgery. Several studies investigated the genetic alteration between the in situ and invasive components of a tumor and found that they share a high degree of similarity^31–34^. Meanwhile, Schuetz et al. investigated the biology of transition from DCIS to IDC and identified progression-specific candidate genes^35^. Those to some extents indicated that the co-existing IDC was evolved from the in situ component, but not due to the intra-tumor heterogeneity. Thus, for tumors with micro-invasive foci that lack sufficient sample to complete the pathological analysis, IHC results from the in situ component can be used for tailoring subsequent treatment.

In the current study, we evaluated factors associated with pathological underestimation in a large cohort of patients diagnosed with pure DCIS at CNB and compared the concordance rate of IHC results between CNB and surgery. However, there were several potential limitations. First, selection bias may serve as an inevitable problem since this was a retrospective study, although we performed multivariate analysis to narrow this effect. Second, with data from a single institute, our results may not be appropriate when being extrapolated to other populations, and multicenter studies warrant consideration. Last but not least, the biopsy method, as well as the number of cores examined, were not considered in the current study. Thus, our results may only have meaning to estimate the upgrade of DCIS by using CNB, and further efforts were needed to evaluate the pathological underestimation by using VAB.

In conclusion, for patients diagnosed with DCIS at CNB, those who had MRI BI-RADS ≥ 5, high nuclear grade, and tumor size ≥ 5.0 cm were more likely to underwent pathological underestimation. ER, PR and HER2 status showed a high concordance rate between CNB and surgery, regardless of the presentation of pathological underestimation.

## Methods

### Patient population

Patients diagnosed with DCIS at biopsy from Jan. 2009 to May. 2020 at Ruijin Hospital, Shanghai Jiaotong University School of Medicine, Shanghai, China were retrospectively reviewed. Data of patients were extracted from Shanghai Jiao Tong University Breast Cancer Database (SJTU-BCDB, registration number of the State Copyright Administration: 2015SR199280), including age at diagnosis, body mass index (BMI), menopausal status, history of benign breast disease, history of malignant disease, family history of breast cancer, clinical symptoms and signs, radiological data, pathological data, as well as immunohistochemical (IHC) results of ER, PR, HER2, and Ki-67. Patients were included if they met the following criteria: (1) received both biopsy and surgery in our center; (2) female gender; (3) pathologically confirmed pure DCIS at biopsy. The following exclusion criteria were applied: (1) pathologically confirmed axillary metastasis preoperatively; (2) had invasive or micro-invasive component confirmed at biopsy; (3) underwent excisional biopsy; (4) had missing data for surgery.

### Clinical and radiological examination

Clinical examination was recorded according to the initial physical examination after hospitalization. Ultrasound imaging examinations used a MyLab 90 ultrasound system (Esaote, Genoa, Italy). Mammography (MMG) imaging examinations were applied a full-field digital flat-panel mammography machine (Senographe DS, GE, USA), allowing for the high-resolution bilateral collection of craniocaudal (CC) and mediolateral oblique (MLO) images. MR imaging examinations were performed by 1.5-T MR imager (Magnetom Aera; Siemens Healthcare, Erlangen, Germany). Imaging assessment was referring to the ACR BI-RADS® Atlas Fourth Edition (before 2013) and ACR BI-RADS® Atlas Fifth Edition (since 2013)^36^. The maximum mass diameter was defined by using ultrasound imaging examination.

### CNB, vacuum-assisted biopsy (VAB), and surgical specimen

The CNB were performed by using 14-gauge (G) automated biopsy guns (Magnum, BARD, Covington, U.S). The VAB were performed by using the Mammotome system with 8-G biopsy guns (Mammotome EX, Devicor Medical Products,Inc., USA) or 13-G biopsy guns (Mammotome elite, Devicor Medical Products,Inc., USA). The procedure was guided by sonographic device (sonosite S-Women’s health). CNB samples were fixed immediately in adequate volume of 4% buffered formaldehyde for at least 6 h according to the American Society of Clinical Oncology/College of American Pathologists (ASCO/CAP) guidelines, and embedded in paraffin for histopathological analysis^37^. The surgical specimen was cut into 1-cm-thick slices, fixed within 1 h, and followed by paraffin embedding.

### Pathological and IHC analysis

Pathological and IHC analyses were conducted at the Department of Pathology, Ruijin Hospital, Shanghai Jiaotong University School of Medicine. Histopathological evaluation was according to the World Health Organization classification^38^. Pure DCIS was recognized if the neoplastic proliferation of epithelial cells was confined to the mammary ductal-lobular system. T1mic referred to DCIS with a microscopic focus of invasion ≤ 0.1 cm in the longest dimension^38^. DCIS + IDC was defined as the presence of both in situ component and invasive component^39^.

The IHC assessment of ER, PR, HER2, and Ki-67 was performed on 4 μm- formalin-fixed, paraffin-embedded tumor samples from CNB sample and surgical specimen, with the following antibodies: ER (SP1, Dako), PR (PgR 636, Dako), HER2 (4B5, Roche), and Ki-67 (MIB-1, Dako). ER-positivity (ER +) and PR-positivity (PR +) were defined as ≥ 1% positive tumor cells with nuclear staining. Ki-67 was characterized as the proportion of positive nuclear staining cells within at least 1000 tumor cells counted^29^. HER2-positivity (HER2 +) was referring to the 2018 ASCO/CAP guidelines, described as 3 + by IHC, or 2 + by IHC and HER2 amplified by fluorescence in situ hybridization (FISH). Cases with 2 + by IHC without results of FISH was regarded as uncertain. The molecular subtype was according to the 2013 St. Gallen breast cancer consensus as follow: Luminal A-like: ER + , PR + , HER2- and Ki-67 < 14%; Luminal B-like (HER2 negative): ER + , HER2-, PR < 20% or/and Ki-67 > 14%. Luminal B-like (HER2 positive): ER + or/and PR + , HER2 + ; HER2-positive (non-luminal): ER-, PR-, HER2 + ; Triple-negative: ER-, PR-, and HER2-^40^. IHC results were assessed based both on the invasive component and in situ component in DCIS + IDC groups.

### Statistical analysis

Categorical variables between patients with and without underestimation were compared by using the Fisher’s exact test. Univariate and multivariate logistic regression analyses were performed to identify clinicopathological characteristics related to pathological underestimation with odds ratio (OR) and 95% confidence interval (CI). Concordance analysis of receptor status and Ki-67 between CNB and surgical specimen was calculated by the kappa test. A κ value > 0.6 is considered as having with good agreement, value between 0.4 and 0.6 suggests moderate agreement, value < 0.4 indicates fair agreement, and value < 0.2 indicates poor agreement. Two-sided *P* values < 0.05 were considered statistically significant. Statistical analyses were carried out by using the IBM SPSS statistics software version 25.0 (SPSS, Inc., IL, USA) and graphing were conducted by GraphPad Prism version 7.0 (GraphPad Software, CA, USA).

### Ethics approval

The study was approved by the Ethical Committees of Ruijin Hospital, Shanghai Jiao Tong University School of Medicine. All procedures performed in studies involving human participants were in accordance with the ethical standards of the institutional and/or national research committee and with the 1964 Helsinki declaration and its later amendments or comparable ethical standards. Informed consent was obtained from all individual participants included in the study.

## Supplementary Information

Supplementary Information.

## Data Availability

The datasets generated during and/or analyzed during the current study are available from the corresponding author on reasonable request.
