# Supplementary material for: Pathological underestimation and biomarkers concordance rates in breast cancer patients diagnosed with ductal carcinoma in situ at preoperative biopsy
Source: Sci Rep. 2022 Feb 9;12:2169. doi: 10.1038/s41598-022-06206-7 (PMC8828849; doi:10.1038/s41598-022-06206-7)
Supplement: Supplementary file 1 — Supplementary Information. [file 41598_2022_6206_MOESM1_ESM.doc]

**Table S1** Concordance between biopsy and surgery for molecular subtypes in patients without pathological underestimation

| **Biopsy of DCIS** | **Surgery of DCIS** | | | | | | **Concordance rate** | **Kappa** | ***P* value** |
| --- | --- | --- | --- | --- | --- | --- | --- | --- | --- |
|  | Luminal A-like | Luminal B-like (HER2 negative) | Luminal B-like (HER2 positive) | HER2 positive (non-luminal) | Triple negative | Total |  |  |  |
| Luminal A-like | 17 | 4 | 1 | 0 | 0 | 22 | 77.3% | 0.684 | <0.001 |
| Luminal B-like (HER2 negative) | 1 | 12 | 1 | 0 | 1 | 15 | 80.0% |  |  |
| Luminal B-like (HER2 positive) | 0 | 2 | 21 | 10 | 0 | 33 | 63.6% |  |  |
| HER2 positive (non-luminal) | 0 | 1 | 10 | 82 | 1 | 94 | 87.2% |  |  |
| Triple negative | 0 | 0 | 1 | 0 | 2 | 3 | 66.6% |  |  |
| Total | 18 | 19 | 34 | 92 | 4 | 167 | 80.2% |  |  |

Including 45 cases unknown in ER PR; 175 cases unknown or uncertain in HER2; 48 cases unknown in Ki-67;

A total of 176 cases unknown in molecular subtype.

Κ values were estimated using kappa test.

Abbreviation: DCIS=ductal carcinoma in situ;

**Table S2** Concordance between biopsy and in situ component at surgery for molecular subtypes in patients with pathological underestimation

| **biopsy of DCIS** | **In situ Component at surgery of IDC** | | | | | | **Concordance rate** | **Kappa** | ***P* value** |
| --- | --- | --- | --- | --- | --- | --- | --- | --- | --- |
|  | Luminal A-like | Luminal B-like (HER2 negative) | Luminal B-like (HER2 positive) | HER2 positive (non-luminal) | Triple negative | Total |  |  |  |
| Luminal A-like | 19 | 11 | 0 | 0 | 0 | 30 | 63.3% | 0.611 | <0.001 |
| Luminal B-like (HER2 negative) | 2 | 17 | 1 | 0 | 1 | 21 | 81.0% |  |  |
| Luminal B-like (HER2 positive) | 1 | 6 | 27 | 20 | 0 | 54 | 50.0% |  |  |
| HER2 positive (non-luminal) | 0 | 2 | 10 | 90 | 2 | 104 | 86.5% |  |  |
| Triple negative | 0 | 4 | 0 | 1 | 11 | 16 | 68.8% |  |  |
| Total | 22 | 40 | 38 | 111 | 14 | 225 | 72.9% |  |  |

51 cases unknown in ER and PR at biopsy; 150 cases unknown in ER and PR at In Situ Component at surgery of IDC; 144 cases unknown or uncertain in HER2 at biopsy; 233 cases unknown or uncertain in HER2 at In Situ Component at surgery of IDC. 47 cases unknown in Ki-67 at Invasive Component at surgery of IDC; 155 cases unknown in Ki-67 at In Situ Component at surgery of IDC.

A total of 167 cases unknown in molecular subtype.

Κ values were estimated using kappa test.

Abbreviation: IDC, invasive ductal carcinoma; CNB, core needle biopsy; DCIS=ductal carcinoma in situ;

**Table S3** Concordance between CNB and invasive component at surgery for molecular subtypes in patients with pathological underestimation

| **CNB of DCIS** | **Invasive Component at surgery of IDC** | | | | | | **Concordance rate** | **Kappa** | ***P* value** |
| --- | --- | --- | --- | --- | --- | --- | --- | --- | --- |
|  | Luminal A-like | Luminal B-like (HER2 negative) | Luminal B-like (HER2 positive) | HER2 positive (non-luminal) | Triple negative | Total |  |  |  |
| Luminal A-like | 10 | 5 | 0 | 0 | 0 | 15 | 66.70% | 0.635 | <0.001 |
| Luminal B-like (HER2 negative) | 3 | 7 | 1 | 0 | 0 | 11 | 63.60% |  |  |
| Luminal B-like (HER2 positive) | 0 | 0 | 19 | 10 | 0 | 29 | 65.50% |  |  |
| HER2 positive (non-luminal) | 0 | 0 | 7 | 44 | 0 | 51 | 86.30% |  |  |
| Triple negative | 0 | 1 | 0 | 1 | 4 | 6 | 66.70% |  |  |
| Total | 13 | 13 | 27 | 55 | 4 | 112 | 75.00% |  |  |

51 cases unknown in ER and PR at biopsy; 44 cases unknown or uncertain in HER2 at biopsy; 170 cases uncertain in HER2 at Invasive Component at surgery of IDC; 47 cases unknown in Ki-67 at Invasive Component at surgery of IDC;

A total of 280 cases unknown in molecular subtype.

Κ values were estimated using kappa test.

Abbreviation: IDC, invasive ductal carcinoma; CNB, core needle biopsy; DCIS=ductal carcinoma in situ

**Table S4** Concordance between in situ and invasive component for molecular subtypes in IDC+DCIS patients

| **In Situ component** | **Invasive component** | | | | | | **Concordance rate** | **Kappa** | ***P* value** |
| --- | --- | --- | --- | --- | --- | --- | --- | --- | --- |
|  | Luminal A-like | Luminal B-like (HER2 negative) | Luminal B-like (HER2 positive) | HER2 positive (non-luminal) | Triple negative | Total |  |  |  |
|  |  |  |  |  |  |  |  |  |  |
| Luminal A-like | 21 | 3 | 0 | 0 | 0 | 24 | 87.5% | 0.820 | <0.001 |
| Luminal B-like (HER2 negative) | 4 | 23 | 0 | 0 | 1 | 28 | 82.1% |  |  |
| Luminal B-like (HER2 positive) | 0 | 1 | 23 | 8 | 0 | 32 | 71.9% |  |  |
| HER2 positive (non-luminal) | 0 | 0 | 0 | 58 | 3 | 61 | 95.1% |  |  |
| Triple negative | 0 | 0 | 0 | 0 | 7 | 7 | 100.0% |  |  |
| Total | 25 | 27 | 23 | 66 | 11 | 152 | 86.8% |  |  |

**^*^**150 cases unknown in ER; **^**^**150 cases unknown in PR; ^#^ 170 cases uncertain in HER2 at Invasive Component at surgery of IDC; 233 cases unknown or uncertain in HER2 at In Situ Component at surgery of IDC; ^##^ 155 cases unknown in Ki-67.

A total of 240 unknown in molecular subtype

Κ values were estimated using kappa test.

Abbreviation: DCIS+IDC, invasive ductal carcinoma with in situ ductal carcinoma component;

**Figure S1** Disconcordance of PR, HER2, and Ki-67 in cases without pathological underestimation


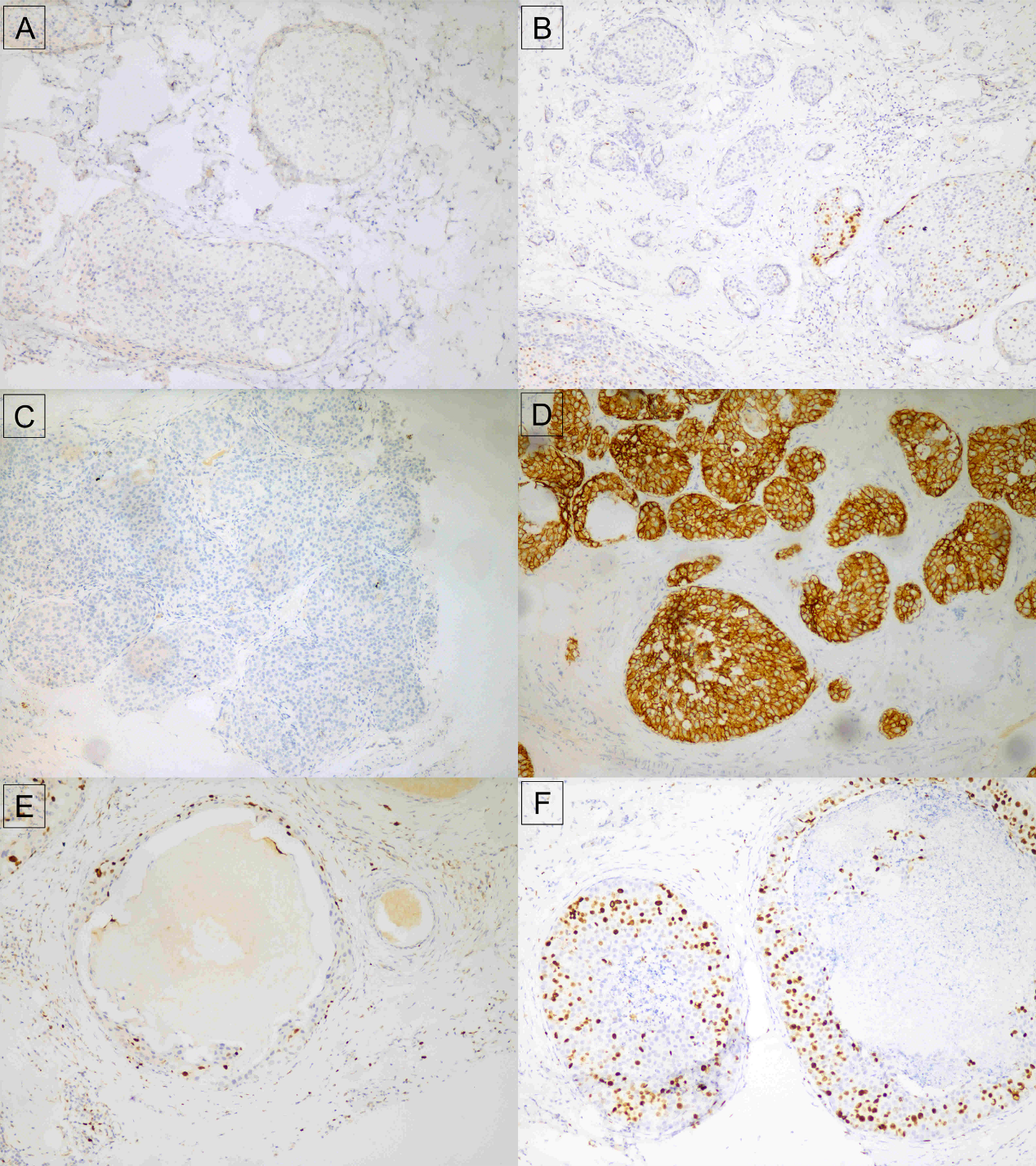


In case 1, the IHC result of PR was negative for biopsy sample (A), which showed weak positive staining for surgery sample (B).

In case 2, the IHC result of HER2 was negative for the biopsy sample (C), which turned to be positive in the surgery sample (C).

In case 3, the IHC result of Ki-67 was low-expressed in the biopsy sample (nearly 10%, E), which was highly expressed in the surgery sample (nearly 40%, F).

**Figure S2** Discordance PR, HER2, and Ki-67 in cases with pathological underestimation


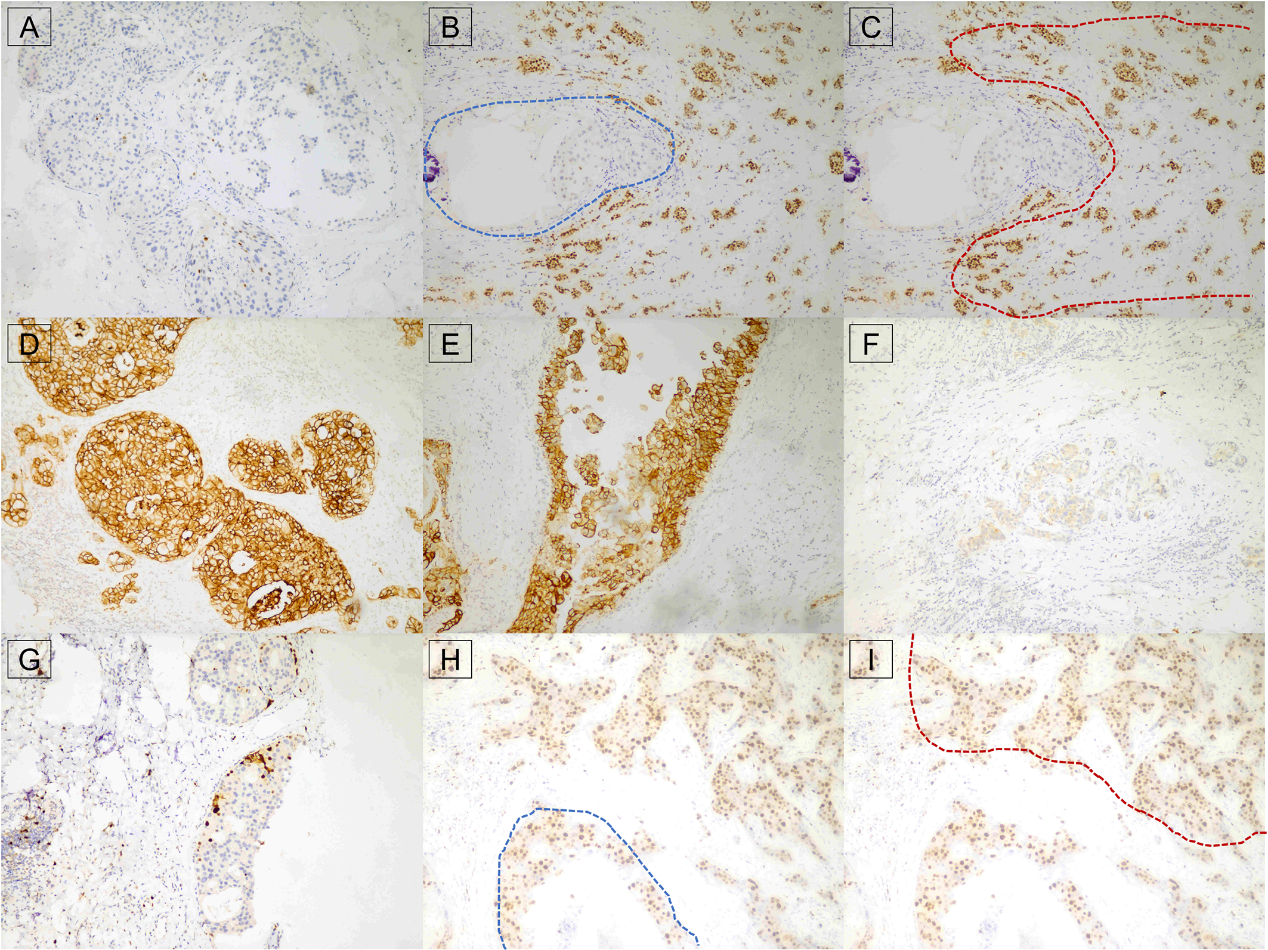


In case 4, the IHC result of PR was negative for biopsy sample (A), which remained negative in the DCIS component (circled with the blue line) in surgery sample (B), but the invasive component was PR positively expressed (circled with the red line) in surgery sample (C).

In case 5, the IHC result of HER2 was positive for biopsy sample (D), which remained positive in the DCIS component in surgery sample (E), while the invasive component in the surgery sample was HER2-negative (F).

In case 6, the IHC result of Ki-67 was low-expressed in the biopsy sample (nearly 10%, G), which was highly expressed in both the DCIS component (circled with the blue line in Figure S3H) and the invasive component (circled with the red line in Figure S3I) in surgery sample.

**Figure S3** Discordance of PR status in synchronous DCIS and IDC


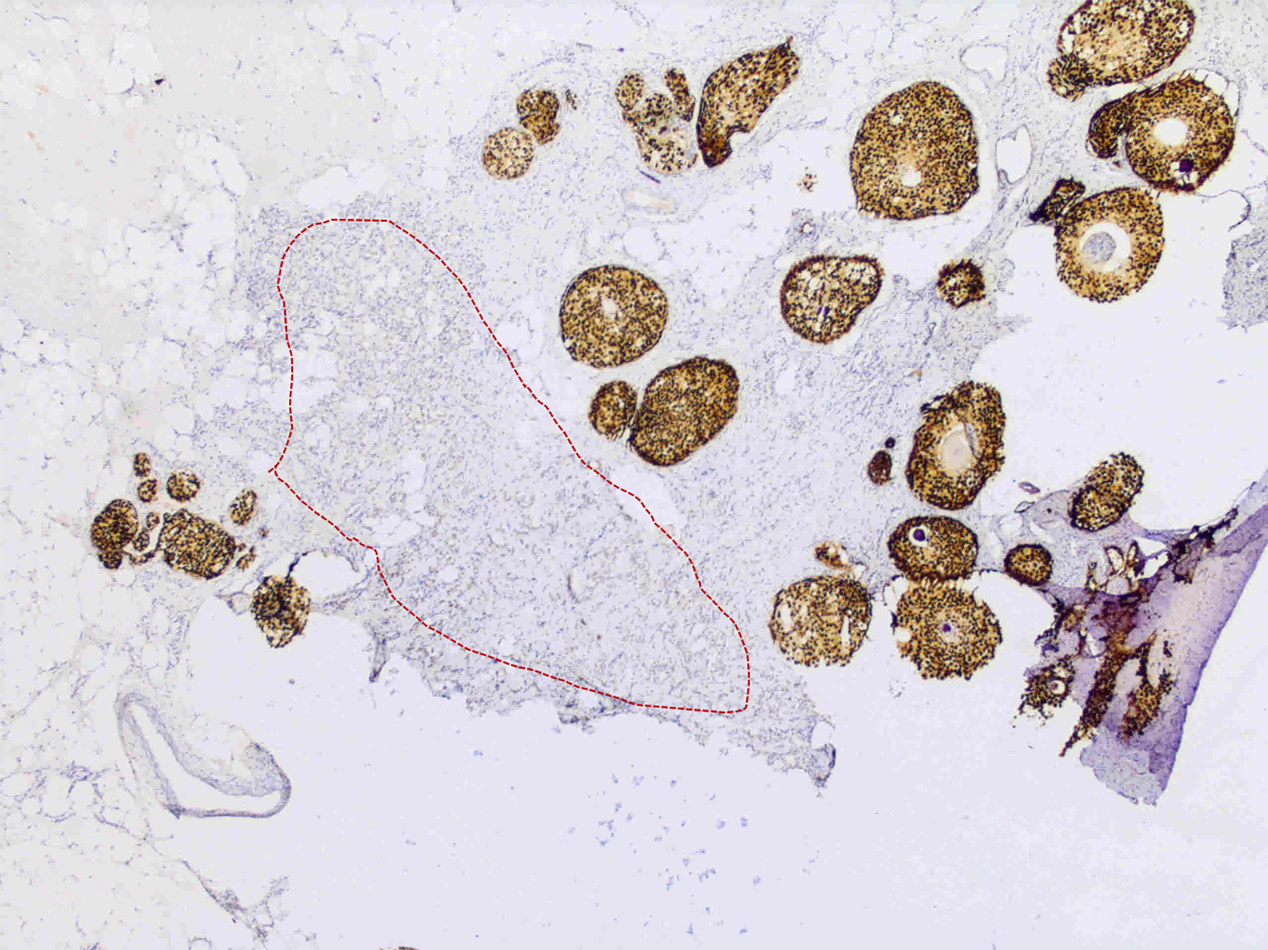


The picture showed the IHC results for ER in synchronous DCIS and IDC within a surgery sample (case 7). The DCIS component showed a strong expression of ER. Whereas, the IDC component, as was circled with the red line, was lack of ER expression.
